# Sentiment analysis of research attention: the Altmetric proof of concept

**DOI:** 10.3389/frma.2025.1612216

**Published:** 2025-10-31

**Authors:** Carlos Areia, Michael Taylor, Miguel Garcia, Jonathan Hernandez

**Affiliations:** ^1^Digital Science, London, United Kingdom; ^2^Department for Intelligent Healthcare, Coventry University, Coventry, United Kingdom; ^3^Statistical Cybermetrics Research Group, Faculty of Arts, Business and Social Sciences, University of Wolverhampton, Wolverhampton, United Kingdom

**Keywords:** AI, sentiment analysis, research attention, discourse, altmetrics, artificial intelligence, LLM

## Abstract

Traditional bibliometric approaches to research impact assessment have predominantly relied on citation counts, overlooking the qualitative dimensions of how research is received and discussed. Altmetrics have expanded this perspective by capturing mentions across diverse platforms, yet most analyses remain limited to quantitative measures, failing to account for sentiment. This study aimed to introduce a novel artificial intelligence-driven sentiment analysis framework designed to evaluate the tone and intent behind research mentions on social media, with a primary focus on X (formerly Twitter). Our approach leverages a bespoke sentiment classification system, spanning seven levels from strong negative to strong positive, to capture the nuanced ways in which research is endorsed, critiqued, or debated. Using a machine learning model trained on 5,732 manually curated labels (ML2024) as a baseline (F1 score = 0.419), we developed and refined a Large Language Model (LLM)-based classification system through three iterative rounds of expert evaluation. The final AI-driven model demonstrated improved alignment with human assessments, achieving an F1 score of 0.577, significantly enhancing precision and recall over traditional methods. These findings underscore the potential of advanced AI methodologies in altmetric analysis, offering a richer, more context-aware understanding of research reception. This study laid the foundation for integrating sentiment analysis into Altmetric platforms, providing researchers, institutions, and policymakers with deeper insights into the societal discourse surrounding scientific outputs.

## 1 Introduction

Measuring the attention and impact that research outputs, such as academic papers, have received has historically been done using citations, and with the advent of digital distribution readership (Thelwall and Kousha, 2015). With the advent of altmetrics (Priem, 2011), a new way of tracking attention using mentions from multiple sources started to emerge as a measure that was arguably (Bornmann, 2014) closer to societal impact (Arroyo-Machado and Torres-Salinas, 2023), complementing citation analysis for academic impact (Costas et al., 2014). These mentions come from news, patents, policy documents, social media posts, blogs, clinical guidelines, and many other sources (Thelwall et al., 2013). Although they have a lot of room to expand (Jarić et al., 2025), they are showing a different way of portraying the attention that research receives, at least in terms of its quality.

Multiple studies assessed the connections between altmetrics and traditional citation metrics, mostly focusing on data such as X (formerly known as Twitter) mentions or Mendeley readers (Sugimoto et al., 2017), but most have been quantitative analyses, focusing on the volume of mentions, rather than qualitative, focusing on what is being said, a deficit that has been recognized for many years (Liu and Adie, 2013). This research provides the foundation for analyzing the context of what is said and its impact on the research within the context of social media mentions. With comprehensive access to social media mentions of research papers (Ortega, 2018), the Altmetric.com database can be analyzed to investigate potential correlations between citations and the qualitative aspects of what is being said, using sentiment analysis.

Traditional sentiment analysis methods often rely on purely lexical approaches, which may struggle with the nuances of academic discourse, sarcasm, and implicit sentiment (Gupta et al., 2024). Large Language Models (LLMs) provide an opportunity to enhance accuracy by considering the full context of a mention, including its relationship with the research being mentioned. With the emergence and development of new AI capabilities, we hypothesized that using a modern LLM, we could have a fitting classification for social media posts mentioning research papers. However, assessing the sentiment of posts demands the addition of a lot of context and prompt optimization to ensure that it is appropriate and well-framed. To the best of our knowledge, no models have focused their sentiment toward the use of research outputs. The primary objective of this study is to confirm the hypothesis that a bespoke AI sentiment analysis can be developed for research on social media and applied at scale. Secondary objectives include exploring whether an LLM can surpass the precision and recall of our older machine learning model.

## 2 Methods

### 2.1 Sentiment analysis rationale and scores

The research attention sentiment analysis was built to target the use of research on social media posts, rather than solely considering the content of the post itself. The objective of the model was to calculate the strength of use, recommendation, and criticism toward research outputs. In this example post, someone replied: “What you are saying makes no sense, and here is the paper to prove it. Doi: xxx.” In a normal sentiment analysis, focusing on its content, we would normally assume the sentiment to be negative, given the negative connotation of the post. However, we aimed to create a sentiment analysis model that would have a positive use of a publication by using it to support a post (even if the content of the post itself is negative). To achieve this, we have tested multiple methods and found out that the best way to calculate this was to create seven levels of sentiment toward the use of research, ranging from a strong negative (−3) to a strong positive (3). Examples can be found in Table 1.

**Table 1:** Bespoke sentiment analysis level with examples.

**Score**	**Example**	**Other types of posts**
Strong Negative (−3)	“This paper is completely biased”	Strong criticism, warning, alerting …
Negative (−2)	“This is preprint so buyer beware but hopefully it holds up.”	Doubt, cautioning, querying …
Neutral negative (−1)	“Oh boy”	Satire, ironic, humor, sad emoji …
Neutral (0)	“https://t.co/u8hSn3x5Lu”	No content
Neutral positive (1)	“COVID-19 diagnosis and management: a comprehensive review (https://t.co/n3WGYvwwHA)”	Title + link, Title + hashtag, happy emoji …
Positive (2)	“New study from Brazil finds” regular use of ivermectin as a prophylactic agent was associated with significantly reduced COVID-19 infection, hospitalization, and mortality rates “(https://t.co/vRjVHAb09s)”	Commentary, suggestion, support, encouragement to read it, sharing results
Strong positive (3)	“Amazing paper”	Recommendation, solution, essential, praise, …

### 2.2 Machine learning sentiment parameters

Our first machine learning sentiment analysis model was created in 2022 (ML2022) and then updated in 2024 (ML2024), both created inside Google Vertex AI AutoML sentiment analysis. These mainly included the content of the post + title of the mentioned publication as context for the sentiment model. ML2022 used 800 manually curated labels individually and was the best among the nine models manually developed and tested at the time. More recently, we evolved this model into a better version (ML2024) that used batch processing for sentiment labeling. This model was trained using 5732 manually curated labels, comprising 4,008 (69.9%) for training, 857 (15.0%) for validation, and 867 (15.1%) for testing. In both models, all posts were manually reviewed by at least two authors and by a third in case of sentiment classification disagreements (e.g., posts that included irony, sarcasm, and ambiguity).

For sentiment prediction, we targeted posts with original content and then automatically labeled all reposts with the same sentiment as their respective original post. Using our old machine learning models since 2022, we have, in total, calculated the sentiment of more than 18 million X posts (Table 2). The latest ML model (ML2024) achieved a precision and recall of 0.418 and an F1 score of 0.419.

**Table 2:** Machine learning sentiment analysis labeled posts.

**Model name**	**Number of original posts**	**Number of reposts**	**Number of all posts**
ML2022	5567350	10793669	16361019
ML2024	727363	1376651	2104014
Totals	6294713	12170320	18465033

### 2.3 AI sentiment

#### 2.3.1 Processing pipeline, prompt, and response

**Figure 1:**
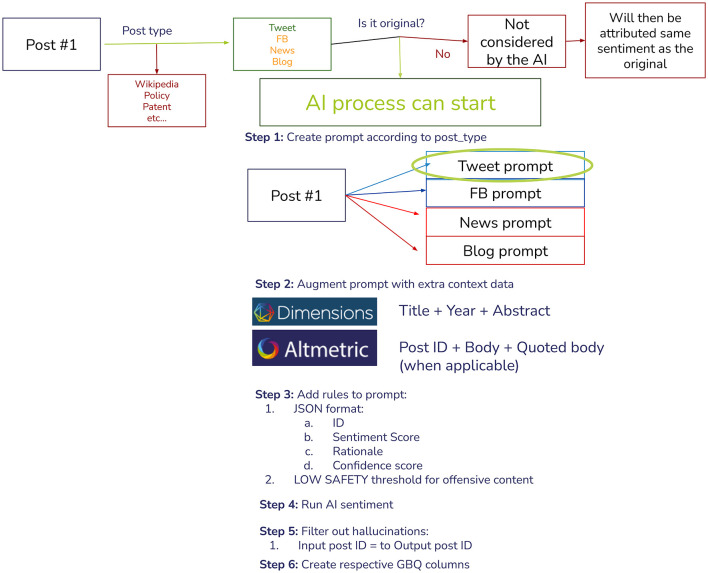
Artificial intelligence sentiment analysis pipeline.

Since Altmetric contains multiple sources, we set up the testing pipeline in a way that would be easy for us to add new sources as Altmetric evolves in the future (for example, Bluesky). For this study, we have focused on X (formerly known as Twitter), but have already conducted some preliminary testing on Facebook, Bluesky, News, and Blogs. The entire pipeline was built, tested, and refined using Google Vertex AI and Google BigQuery (Figure 1).

The first step was to extract all posts of interest and filter the eligible ones for the AI model (X original post). We would then link all relevant Dimensions and Altmetric data for that post to be used in the prompt. In this, we would then request the AI to return a JSON with the following fields:

- Publication ID: To filter hallucinations, our first step after the response was to link only the responses that provided a valid Publication ID back
- Sentiment score
- Rationale: To allow for iteration and to better understand any potential patterns and bias in the prompt or response
- Confidence Score: How confident was the AI that it returned the correct response? This score was not accurate; therefore, we did not use it in this study.

We then used the Gemini 1.5 Flash model with the temperature set to 0.2 and a Low safety threshold to allow offensive content to be analyzed. Our prompt would look something like this:

*You are a researcher and social media attention specialist in the label of sentiment analysis of tweet/X toward the use of a publication or research output. The tweet always tags a research paper. Your role is to label from* −*3 (strong negative) to 3 (strong positive)*.

*SA scores:*

−*3 Strongly negative, e.g., warning, alert, strong criticism of cited paper. For example, when someone openly critiques the publication (“So much wrong with this horrible study: A 4-Day Mindfulness-Based Cognitive Behavioral Intervention Program for CFS/ME. An Open Study, With 1-Year Follow-Up;* *https://t.co/NOg1ZAxGQ9* *; #MEcfs #pwME”)*.
*-2 Moderate negative, e.g., casting doubt, querying, questioning, cautioning. For example, when one can see it's negative but there is not a lot of context around (“I fell off the chair laughing on this one!!! 7* 
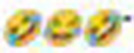
”* or “Ooops…they did it again* 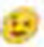 *#covid19”)*
*-1 Uncertain negative, e.g., satire, ironic, humor, concern, scared. For example, when there is a small hint that it might be negative but very unclear, or just particular emojis (“@NutriDetect is telling me Vit D is killing me. What?” or “Can we stop calling it a conspiracy now?” or “*
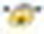
”* or “uh oh”)*
- *0 Neutral, contains no content. For example, when someone just shares the link, or just the title and link for the study*
- *1 Uncertain positive, e.g., title and link from a human. For example, when someone just posts the link or title and link, but tags someone or uses hashtags (“Original Article from The New England Journal of Medicine”)*
- *2 Moderate positive, e.g., title, link, some commentary, have you seen this, reading suggestion, might be useful. For example, when someone shares the conclusions or results of the study, recommends reading the publication, congratulates the team for the work, or says it is interesting, or uses it to support an argument/claim without explicitly praising the paper*.
- *3 Strong positive, e.g., congratulations, recommendation, read this, essential, solution, etc. For example, when someone posts amazing work, a great study, provides evidence or proof, or says it is a must-read, etc*.

*To help with your decision-making, I have included the publication title, abstract, and year, allowing you to review and assess the context and decide whether the body of the post refers to the topic positively or negatively*.

*When the body is quoting a tweet, this is also provided for context*.

*Please review it carefully and use your judgment to give a thoughtful conclusion. Using this information, label the sentiment only of the tweet content (that is, between brackets [])*.

*Output format: Answer in JSON format with four keys: “ID” as a string, “score” as a string, “confidence” as a number, and “explanation” as a string. Always start with the “ID” first, then the “sentiment” score of the analyzed post in second, then your “confidence” score that the sentiment is correct (on a scale from 0 to 1, be conservative and really quantify your confidence in your sentiment score) in third, and finally the “explanation”*.

*A few things to note:*

1. *Only score the body of the tweet (inside brackets [] only). All the rest should be used for context only. Familiarize well with all the context before conducting the sentiment analysis*.
2. *Check if the majority of the tweet is mentioning the title and/or abstract of the publication (review each field and compare with the body). If it is, then the sentiment is unclear positive (1). Where the quoted tweet is available, use it for context as well*.
3. *Only score the sentiment toward the use of the research publication, not the content, wording, or language of the tweet itself. Please make sure you score it according to how the publication is being mentioned, not the content of the tweet (a negative tweet can have neutral/positive sentiment in this model if it uses the research paper neutrally or positively)*.
4. *If the content of the tweet doesn't seem to explicitly talk about research, assume it is either unclear, positive, or negative because it mentions and uses the publication (even if just the link)*.
5. *When in doubt, lean toward unclear positive/negative (for example, when posts are very positive or negative but not toward the publication). Keep it between* −*1 and 1 whenever you are questioning the sentiment toward the research publication. Always be conservative in your sentiment*.
6. *Be careful when the content of the post seems to be negative (sometimes they can be using the publication to back up their claim or refuting someone else's claim in their reply, even if it's negative, so that would be a positive use of the research). Double-check all* −*2 or* −*3 scores to make sure it's clearly a negative post*
7. *Also watch out for satire, cynicism, and irony*.
8. *Double-check if the content of the post is the same or similar to the title or sentences inside the abstract; if that is the case, the score should be toward neutral (between* −*1 and 1)*.

*Here is the ID of the post: Post ID*

*Quoted post: Quoted post body*

*Content: Post Body*

*Publication title: Publication Title*

*Year: Publication Year*

*Abstract: Publication Abstract*

**Figure 2:**
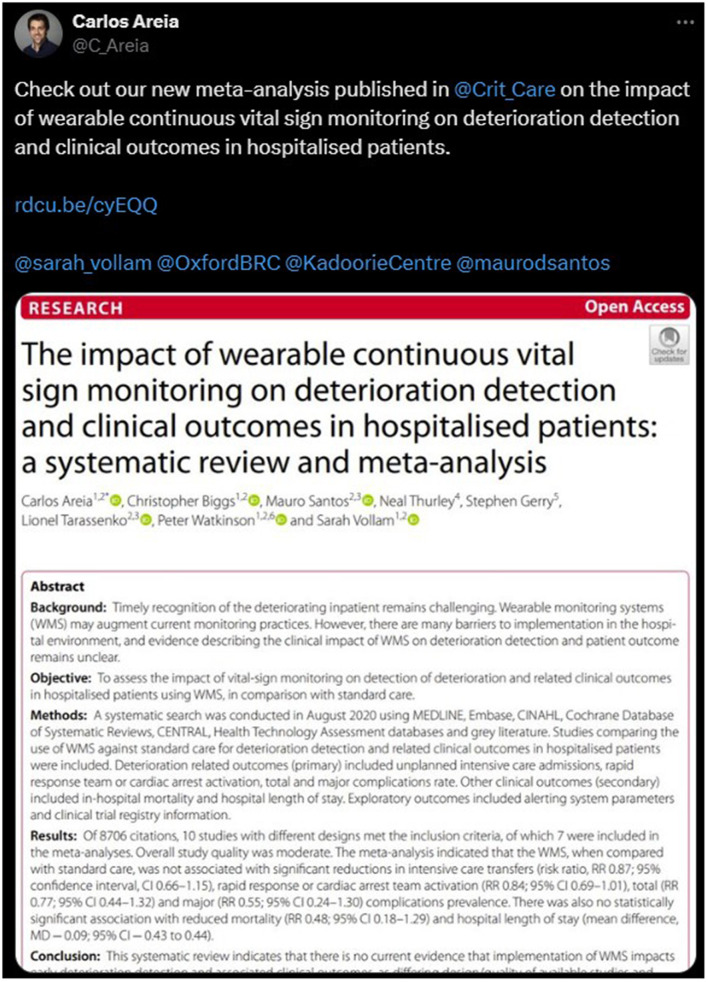
Example post (shared with author's permission).

If we use this publication and post (http://twitter.com/C_Areia/statuses/1443169902983012357, Figure 2) as an example (shared with the author's permission), the content would look something like this inside our prompt:

*Content: [Check out our new meta-analysis published in @Crit_Care on the impact of wearable continuous vital sign monitoring on deterioration detection and clinical outcomes in hospitalized patients*.

*https://t.co/qSCIzUAPws*

@*sarah_vollam @OxfordBRC @KadoorieCentre @maurodsantos;* *https://t.co/ppHattcnKS* *]*

*Publication title: The impact of wearable continuous vital sign monitoring on deterioration detection and clinical outcomes in hospitalized patients: a systematic review and meta-analysis*

*Year: 2021*

*Abstract: Objective: To assess the impact of vital-sign monitoring on the detection of deterioration and related clinical outcomes in hospitalized patients using WMS, in comparison with standard care. Methods: A systematic search was conducted in August 2020 using MEDLINE, Embase, CINAHL, Cochrane Database of Systematic Reviews, CENTRAL, Health Technology Assessment databases, and gray literature. Studies comparing the use of WMS against standard care for detecting deterioration and related clinical outcomes in hospitalized patients were included. Deterioration-related outcomes (primary) included unplanned intensive care admissions, rapid response team or cardiac arrest activation, and total and major complications rate. Other clinical outcomes (secondary) included in-hospital mortality and hospital length of stay. Exploratory outcomes included alerting system parameters and clinical trial registry information*.

*Results: Of 8706 citations, 10 studies with different designs met the inclusion criteria, of which 7 were included in the meta-analyses. Overall study quality was moderate. The meta-analysis indicated that the WMS, when compared with standard care, was not associated with significant reductions in intensive care transfers (risk ratio, RR 0.87; 95% confidence interval, CI 0.66–1.15), rapid response or cardiac arrest team activation (RR 0.84; 95% CI 0.69–1.01), total (RR 0.77; 95% CI 0.44–1.32) and major (RR 0.55; 95% CI 0.24–1.30) complications prevalence. There was also no statistically significant association with reduced mortality (RR 0.48; 95% CI 0.18–1.29) and hospital length of stay (mean difference, MD – 0.09; 95% CI – 0.43 to 0.44).Conclusions: This systematic review indicates that there is no current evidence that implementation of WMS impacts early deterioration detection and associated clinical outcomes, as differing design/quality of available studies and diversity of outcome measures make it difficult to reach a definite conclusion. Our narrative findings suggested that alarms should be adjusted to minimize false alarms and promote rapid clinical action in response to deterioration. PROSPERO Registration number: CRD42020188633*.

#### 2.3.2 Volunteer evaluation workflow

Thirteen volunteers were asked to pick their favorite publications to be included in the testing pipeline, which included the following steps:

1. Participants selected their favorite publications
2. The final publication list was compiled and linked to all respective X posts mentioning each publication using the Altmetric database
3. Ran the aforementioned AI sentiment analysis pipeline (as described in Figure 1)
4. Created an AI table with results and sent it back to volunteers for review, fields described in Table 3
5. Participants were asked to complete the fields agree, name, manual_score, and feedback for all posts linked to the publications of their choosing (as per step 1) up to the deadline established by the authors
6. Once the deadline has been reached, authors closed access to the spreadsheet, analyzed participants' agreement with AI sentiment, their feedback, and refined the prompt.
7. Repeated steps 1–6 until participants' feedback indicates the majority was happy with the results.

**Table 3:** Volunteer spreadsheet fields for AI sentiment analysis, agreement and feedback through iterations.

**Field**	**Description**	**Example**
Tester	Volunteer name linked to that publication	Carlos
doc_id	Dimensions publication ID	pub.1141444795
post_id	Altmetric post ID	518239159
post_type	Altmetric post type (for the purposes of this study only *tweet* was included)	tweet
post_body	Post content	“Check out our new meta-analysis published in @Crit_Care on the impact of wearable continuous vital sign monitoring on deterioration detection and clinical outcomes in hospitalized patients. https://t.co/qSCIzUAPws; @sarah_vollam @OxfordBRC @KadoorieCentre @maurodsantos; https://t.co/ppHattcnKS”
post_url	X post url	http://twitter.com/C_Areia/statuses/1443169902983012357
Sentiment	AI sentiment response	2
sentiment_explanation	AI reasoning for sentiment score	“The tweet is promoting the publication and highlighting its findings. It mentions the publication's title and provides a link to it. The tweet also tags relevant organizations and individuals, suggesting a positive sentiment toward the research.”
sentiment_confidence	AI confidence in the given sentiment	0.9
pub_title	Publication Title	The impact of wearable continuous vital sign monitoring on deterioration detection and clinical outcomes in hospitalized patients: a systematic review and meta-analysis
pub_abstract	Publication Abstract	“Background Timely recognition of the deteriorating inpatient remains challenging. Wearable monitoring systems (WMS) may augment current monitoring practices. However, there are many barriers to implementation in the hospital environment, and evidence describing the clinical impact of WMS on deterio-ration detection and patient outcome remains unclear. Objective to assess the impact of vital-sign monitoring on detection of de-terioration and related clinical outcomes in hospitalized patients using WMS, in comparison with standard care. Methods A systematic search was conducted in August 2020 using MEDLINE, Embase, CINAHL, Cochrane Database of Systematic Reviews, CENTRAL, Health Technology Assessment databases and gray literature. Studies comparing the use of WMS against standard care for deterioration detection and related clinical outcomes in hospitalized patients were included. Deterioration related outcomes (primary) included unplanned intensive care admissions, rapid response team or cardiac arrest activation, total and major complications rate. Other clinical outcomes (secondary) included in-hospital mortality and hospital length of stay. Exploratory outcomes included alerting system parameters and clinical trial registry information. Results Of 8706 citations, 10 studies with different designs met the inclusion criteria, of which 7 were included in the meta-analyses. Overall study quality was moderate. The meta-analysis indicated that the WMS, when compared with standard care, was not associated with significant reductions in intensive care transfers (risk ratio, RR 0.87; 95% confidence interval, CI 0.66–1.15), rapid response or cardiac arrest team activation (RR 0.84; 95% CI 0.69–1.01), total (RR 0.77; 95% CI 0.44–1.32) and major (RR 0.55; 95% CI 0.24–1.30) complications prevalence. There was also no statistically significant association with reduced mortality (RR 0.48; 95% CI 0.18–1.29) and hospital length of stay (mean difference, MD-−0.09; 95% CI-−0.43 to 0.44).Conclusion This systematic review indicates that there is no current evidence that implementation of WMS impacts early deterioration detection and associated clinical outcomes, as differing design/quality of available studies and diversity of outcome measures make it difficult to reach a definite conclusion. Our narrative findings suggested that alarms should be adjusted to minimize false alarms and promote rapid clinical action in response to deterioration.PROSPERO Registration number: CRD42020188633.”
pub_altmetric_id	Publication Altmetric ID	114215230
pub_altmetric_score	Publication Altmetric Score	39
Agree	Volunteer agree/disagree with AI sentiment (Yes/No)	Yes
Name	Volunteer name	Carlos
manual_score	Manual score in case of disagreement with AI	*null*
Feedback	Feedback (mostly used in case of disagreement)	*null*

#### 2.3.3 Data analysis

To calculate improvements between rounds, the difference between AI-volunteer sentiment level was calculated at the post level (for example, if AI sentiment was labeled as 3 and the volunteer disagreed, with a manual sentiment of −1, the difference was 4). All posts with at least one volunteer labeled agreement or disagreement (and when in disagreement, provided with the human volunteer score) served as the ground of truth for all accuracy metrics (Appendix 1).

Descriptive statistics will be shown in mean and standard deviation (Mean ± SD) or percentages. To evaluate classification performance, we will report precision, recall, and F1 score for each sentiment class. Precision measures the proportion of predicted sentiment labels that were correct, while recall reflects the proportion of true sentiment labels that were correctly identified. The F1 score is the harmonic mean of precision and recall, and balances the two in a single metric. These metrics are particularly important in our multi-class ordinal sentiment task, where some sentiment levels (e.g., strongly negative) are underrepresented. Unlike accuracy, which may be skewed by class imbalance, precision and recall allow us to assess how well the model identifies minority sentiment categories and whether it tends to over- or under-predict specific classes. Given the ordinal nature of our labels, small misclassifications (e.g., from −2 to −1) are less problematic than polarity flips (e.g., from −3 to +2), and the F1 score provides a useful summary of this classification quality across sentiment levels. We have also performed a bucket analysis by collapsing the 7-point sentiment scale into three categories—negative (−3 to −2), neutral (−1 to 1), and positive (2 to 3)—to evaluate broader sentiment polarity and reduce sensitivity to minor ordinal mismatches. Jupyter Notebook, Python, and Google Sheets were used in all analyses.

## 3 Results

The study aimed to assess whether AI can be used for sentiment analysis of research mentions at scale and whether it improves upon our existing machine_learning model. For this, we conducted three rounds of iterations with the 13 volunteers between July and September 2024; more information is described in Table 4. Comparing the level of agreement between the AI and the volunteers between iterations has evolved from 0.564 ± 0.956 in round 1, to 0.242 ± 0.696 in round 2, and 0.237±0.813 in round 3, as well as the percentage of overall agreement (64.3%, 85.0%, and 87.2%, respectively), as shown in Table 5.

**Table 4:** AI prompt refinement round metrics.

**Metric**	**Round 1**	**Round 2**	**Round 3**
Number of volunteers included	13	13	18
Number of volunteers contributing	8	7	7
Number of publications included	103	103	354
Number of publications analyzed	36	34	90
Number of posts included	6,887	6,782	15,720
Number of posts analyzed	297	972	618

**Table 5:** Frequency of sentiment difference between the AI result and the volunteer.

**Sentiment difference (AI and volunteer)**	**Round 1**	**Round 2**	**Round 3**
0 (agreed)	191 (64.3%)	826 (85.0%)	539 (87.2%)
1	71 (23.9%)	71 (7.3%)	44 (7.1%)
2	14 (4.7%)	38 (3.9%)	10 (1.6%)
3	13 (4.4%)	14 (1.4%)	3 (0.5%)
4	6 (2.0%)	11 (1.1%)	8 (1.3%)
5	1 (0.3%)	0 (0.0%)	8 (1.3%)
6	0 (0.0%)	0 (0.0%)	0 (0.0%)

Cell background color encodes the magnitude of the AI-volunteer difference: green = agreement (0–1); warmer colors indicate larger differences (2–6).

**Figure 3:**
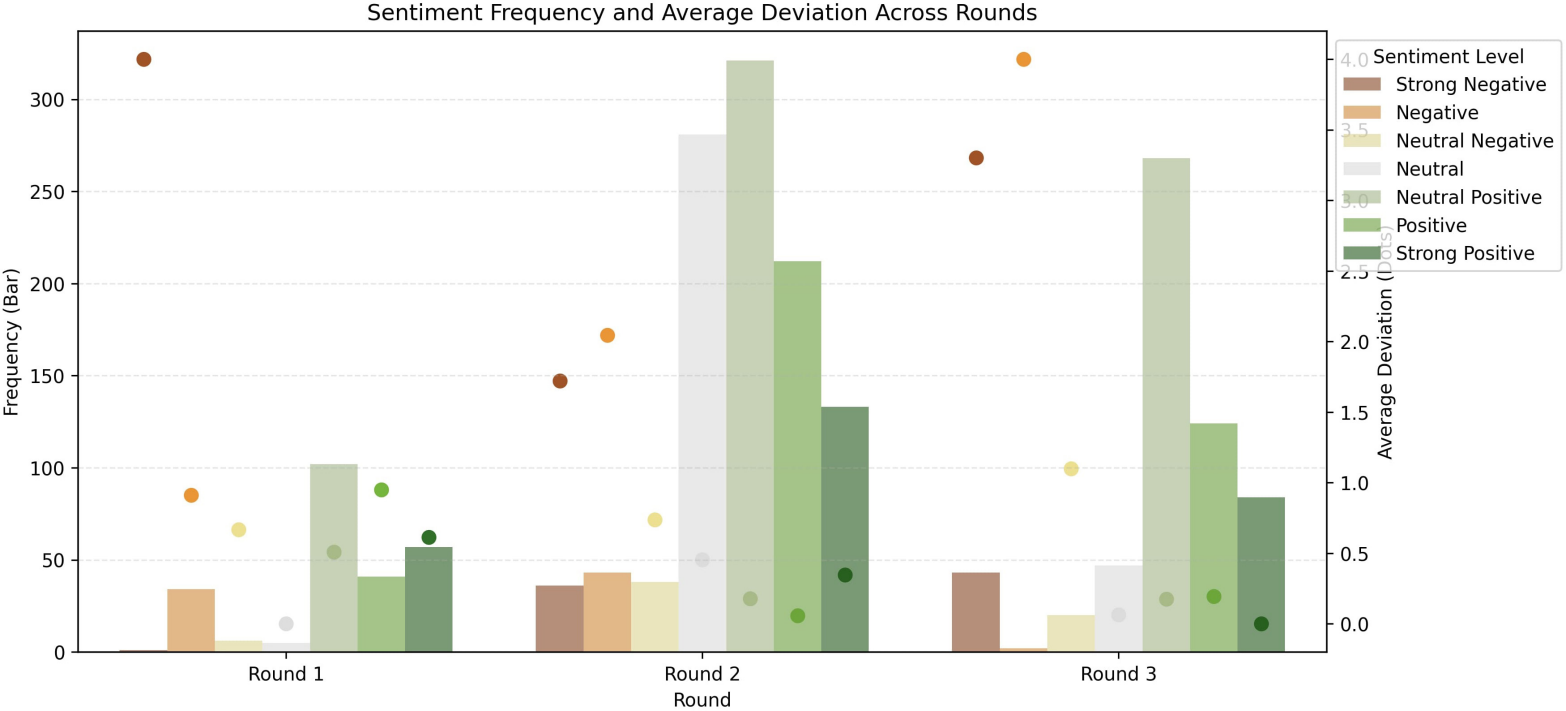
Frequency and average difference between AI model and volunteers on each sentiment level through rounds.

A more granular analysis of the frequency and sentiment differences between the AI model and volunteers can be explored in Figure 3. Here, it is possible to see the general improvement between rounds, especially rounds 1 and 2, at most sentiment levels, especially strong negative and all positive levels. In round 3, there was still noticeable improvement in the average deviation between AI sentiment and volunteer manual scoring for neutral and positive sentiments, and a decline in negative sentiment. Some improvements in precision and recall were observed primarily between rounds 1 and 2, with a slight decline in the F1 score between rounds 2 and 3 (Table 6). To better understand the round 3 model's practical performance, we reclassified the original 7-point sentiment labels into three broader sentiment buckets: negative (−3 and −2), neutral (−1, 0, 1), and positive (2 and 3). This categorization allowed us to assess whether seemingly large errors (e.g., a −3 prediction when the true label is −1) still reflected the correct overall sentiment polarity. Under this bucketed scheme, the Round 3 model achieved an accuracy of 87.2% and a weighted F1 score of 0.89, a substantial improvement over the original fine-grained F1 score of 0.577. This indicates that most discrepancies in the 7-point evaluation are minor ordinal shifts rather than true polarity errors. Class-wise performance in this scheme was (confusion matrix available in Table 7):

- Positive sentiment: F1 score = 0.88 (Precision = 0.97, Recall = 0.80)
- Neutral sentiment: F1 score = 0.91 (Precision = 0.90, Recall = 0.93)
- Negative sentiment: F1 score = 0.37 (Precision = 0.24, Recall = 0.73)

**Table 6:** Precision, recall, and F1 score through rounds.

**Model/iteration**	**Precision**	**Recall**	**F1-score**
ML2024	0.418	0.418	0.419
AI round 1	0.493	0.421	0.397
AI round 2	0.630	0.666	0.635
AI round 3	0.579	0.724	0.577

**Table 7:** Round 3 bucketed confusion matrix.

**Actual/ Predicted**	**Predicted negative (−3 and −2)**	**Predicted neutral (−1, 0 and 1)**	**Predicted positive (2 and 3)**
Actual negative (−3 and −2)	11	4	0
Actual neutral (−1, 0 and 1)	16	300	6
Actual positive (2 and 3)	18	31	202

### 3.1 Narrative analysis of LLM sentiment explanation and volunteer feedback

The explanations provided by the LLM for each sentiment classification offer insights into how evaluators determined sentiment scores, how posts interacted with research, and the implications of these classifications. We conducted a rapid narrative analysis per sentiment level and found that posts classified as strongly negative (−3) exhibit explicit disapproval or criticism toward the research publication. These explanations often reference negative reactions to the findings, expressions of disbelief, or accusations against the integrity of the research. Common terms in these explanations include “strong negative sentiment,” “criticizes findings,” and “opposes conclusions.” This category is relatively small but represents the most intense disagreements or criticisms found in the dataset. The explanation for negative posts included expressing skepticism or disagreement, but did not entirely dismiss the research. Explanations for these posts often highlight doubt in the methodology, displeasure with the results, or concerns about bias. Frequent words in these explanations include “negative sentiment,” “expresses concerns,” and “suggests flaws.” Unlike −3 posts, these posts do not attack the research directly but rather question its reliability or relevance. Posts in the Neutral Negative (−1) category tend to be neutral or slightly negative, often misrepresenting research findings or lacking context. Sentiment explanations often describe these as “potentially misleading,” “misinterprets results,” or “suggests an unintended meaning.” The inclusion of a research link alone without substantive commentary could also contribute to a negative classification if the post's framing is suggestive of doubt or indirect critique. Neutral sentiment (0) was primarily assigned to posts that provide a factual mention of the publication without opinionated language. Explanations for these cases frequently described tweets as simply sharing a link, listing co-authors, or providing a direct title without additional commentary. The most common terms in this category are “neutral sentiment,” “does not express opinion,” and “provides information without bias.” Neutral positive posts (1) expressed some level of endorsement for the research. Explanations often cited phrases like “recommends the publication,” “mentions findings in a constructive way,” and “suggests value.” These posts may briefly praise the research but lack strong enthusiasm. Positive sentiment (2) included posts that share the publication with enthusiasm, provide supportive commentary, or highlight important findings. Common phrases in explanations include “expresses support,” “emphasizes significance,” and “praises findings.” These posts often include research links along with positive framing or personal endorsement. Strongly positive (3) posts reflect high enthusiasm or endorsement of the research. Sentiment explanations often reference explicit praise, high-impact retweets from authoritative sources, or direct statements about the importance of the study. Key words include “strongly recommends,” “highly significant,” and “major breakthrough.” These posts serve as powerful signals of engagement and interest in academic discourse.

The analysis of sentiment classification disagreements revealed several recurring issues that affected the accuracy of sentiment analysis, which supported iterations and improvements between rounds. A primary source of disagreement stemmed from the misinterpretation of language, particularly in non-English tweets, where the LLM failed to account for linguistic nuances. Additionally, it sometimes struggled with contextual subtleties such as sarcasm, implicit criticism, and mixed sentiments, leading to misclassifications highlighted by the volunteers. Posts containing only links or minimal text also posed challenges in round 1, as the model tended to infer sentiment despite a lack of explicit opinion. This misalignment between automated and manual scoring suggested that the model relied heavily on keyword matching rather than contextual understanding. The sentiment explanations provided by the model further reinforced these issues, demonstrating a reliance on individual words such as “positive,” “important,” or “exciting,” while failing to detect sarcasm, negations, or nuanced scientific language. This significantly improved after the model was improved to Gemini 1.5 Flash in round 2, and the quoted context was added in round 3.

## 4 Discussion

In 2022, we decided to create a bespoke machine learning model due to the lack of existing sentiment analysis models targeting the sentiment of research outputs. As AI started to evolve, we kept a close eye on opportunities to upgrade and improve our current model. Despite its imperfections, our ML model's precision, recall, and F1 score accurately reflected the difficulties inherent in quantifying research engagement on social media. These challenges include the subjective nature of categorizing sentiment on a 7-point scale and the overall complexity of understanding how individuals use research within a social media context. With the rapid evolution of LLMs, we quickly realized that it could easily capture irony, sarcasm, and other post/mention content with more nuanced indications and ambiguities that our old ML model was not able to capture, in line with previous literature on the growth of AI and sentiment analysis (Miah et al., 2024).

The primary upgrade between the 1st and 2nd rounds was the addition of quoted tweets, which provided further context and instantly increased the quality of our AI model. Between rounds 2 and 3, we also upgraded our model from Gemini 1.0 to Gemini 1.5 Flash, besides some minor prompt refinements. During the review of round 3 results, volunteers agreed that we reached a plateau of improvement for our AI sentiment analysis proof of concept work. The agreement was high from the outset, considering all posts where sentiment was agreed by the AI and the volunteer, or had a marginal difference of 1. For example, where the LLM assigned a sentiment of 2 (Positive), and the volunteer assigned a 3 (Strong Positive). The majority of posts, 94.3%, fell within this range by round 3. However, we did notice a decline in performance of the correct labeling of strong negative posts in round 3, one of the reasons being the addition of new and more controversial publications (Sullivan and Hickel, 2023; Price et al., 2023; Hanna et al., 2022) to make it more challenging for the LLM under test and this was reflected in the model performance.

Nonetheless, while the F1 score slightly decreased in Round 3, recall improved significantly. This outcome is particularly relevant for sentiment analysis tasks that involve multi-level sentiment classification. The increase in recall suggests that the model is now more effective at capturing a broader range of sentiments, even if precision is slightly reduced (Alsagri and Sohail, 2024). A higher recall means that fewer true sentiment cases are missed, ensuring that strongly positive or negative expressions are correctly identified rather than misclassified as neutral. This is particularly beneficial for sentiment analysis applications in opinion mining, feedback analysis, and general social media monitoring, where failing to detect sentiment-laden expressions could lead to biased or incomplete insights. In contrast, a slight drop in precision (which contributed to the reduced F1 score) indicates that some sentiment predictions might be misclassified into neighboring sentiment levels, but they remain within the general spectrum of positive, neutral, or negative sentiment. This trade-off is often desirable in sentiment analysis as it is inherently subjective, and misclassifications between adjacent levels (e.g., slightly positive vs. neutral) are less critical than failing to detect sentiment altogether. By capturing a greater number of sentiment-laden cases, the model reduces the risk of overlooking significant trends in sentiment analysis. Considering this, and to complement the fine-grained evaluation, we also analyzed model performance using a coarser sentiment bucket scheme, grouping scores into negative (−3 and −2), neutral (−1 to 1), and positive (2 and 3). This approach yielded a notably higher F1 score (0.89) and overall accuracy (87.2%) in Round 3, suggesting that most classification discrepancies occur between adjacent sentiment levels rather than across polarity boundaries. These findings reinforce that the LLM reliably captures the general direction of sentiment, and that many so-called errors on the 7-point scale represent ordinal proximity rather than substantive misclassification. Despite the strong performance of our LLM sentiment classification model, our narrative analysis revealed key areas for future improvement that could further enhance its accuracy. The model effectively captured sentiment in most cases, but challenges remained in handling nuanced language, such as sarcasm, implicit criticism, and mixed sentiments, which were improved through the rounds but still have more room for development. These are common challenges to LLM sentiment analysis (Kheiri and Karimi, 2023).

Prior studies have investigated sentiment analysis of tweets referencing scientific publications, often using lexicon-based or machine learning approaches. (Hassan et al. 2020) applied a domain-adapted version of SentiStrength to over 6.4 million tweets linked to research outputs, identifying variation in sentiment across disciplines and noting higher negative sentiment in health-related fields. (Shahzad and Alhoori 2021) used both VADER and TextBlob libraries alongside Random Forest classifiers to predict tweet sentiment for 148,000 articles, achieving up to 89% accuracy in binary classification. Their models emphasized structural features such as title sentiment, author count, and follower reach as the primary drivers of prediction. While these studies demonstrated scalable sentiment classification pipelines, they focused on general emotional polarity and lacked contextual understanding of how the cited research was used or referenced. In contrast, our study introduces a bespoke seven-level sentiment taxonomy that explicitly distinguishes sentiment toward the use of research outputs, rather than the content or tone of the post itself. This richer framing enables more nuanced interpretation, such as classifying a sarcastic post that uses a paper to challenge a point as positive toward the research, even when surface-level sentiment might appear negative. Compared to (Bichara et al. 2022), who analyzed public engagement with science via NOS frameworks during COVID-19 using keyword-based classification, our approach enables scalable automation across altmetric platforms without requiring manual tweet selection or coding. Furthermore, despite not noticing a significant difference in model performance from Gemini 1.0 to 1.5 Flash, we set up the future pipeline to constantly test, evaluate, and implement newer and better models as AI continues to improve.

Although several recent studies comparing AI to humans on sentiment analysis of social media posts with similar accuracy results (Ahmad et al., 2025; Kim and Kim, 2025; Kim et al., 2024) to the best of our knowledge, this is the first study examining LLM led sentiment analysis of research output mentions, using both post and publication data as context, and planned to be done at scale (>250 million posts). Our proof of concept demonstrates that integrating an LLM-based sentiment analysis framework significantly improves both precision and recall over our earlier machine learning models. This study also provides a first step in addressing a long-standing limitation in alternative metrics research- its over-reliance on quantitative indicators such as mention counts, without considering the context or tone of those mentions (Liu and Adie, 2013; Sugimoto et al., 2017). By integrating sentiment analysis specifically tailored to how research outputs are used or cited in social media discourse, this work offers a methodological advancement for measuring societal attention. The introduction of a bespoke, multi-level sentiment classification scheme, optimized for the nuances of academic mentions, opens new pathways for understanding the qualitative reception of research beyond traditional citations (Arroyo-Machado and Torres-Salinas, 2023). Moreover, by applying LLMs capable of handling sarcasm, irony, and implicit meaning—linguistic features often missed by legacy machine learning or lexical models (Gupta et al., 2024). This study helps bridge the gap between technical innovation in NLP and the applied needs of scientometrics. The resulting framework enhances the interpretability of altmetric indicators, enabling scholars and evaluators to better distinguish between positive engagement, critical scrutiny, or neutral referencing, each of which carries different implications for impact assessment (Costas et al., 2014). This approach not only supports a richer conceptualization of research visibility but also aligns with ongoing calls to democratize and contextualize scholarly metrics (Jarić et al., 2025), making it a timely contribution to the field.

### 4.1 Limitations

There are still several improvements to be made to our LLM sentiment; however, we believe we have achieved an acceptable level of quality and a significant improvement over our older ML model from this proof of concept work. A current LLM limitation observed throughout all rounds was double-negative posts that could give inconsistent or wrong results when tested multiple times with the same prompt. Another challenge that we will need to address in the future is bias toward retracted publications, which currently defaults to more negative sentiment in some particular cases.

Moreover, the selection of included publications was entirely arbitrary according to our volunteers' interests and preferences. Despite the good diversity in our dataset, some bias toward clinical and medical sciences might affect the generalizability of our results. We will investigate this and other potential biases in future iterations.

### 4.2 Future work

**Figure 4:**
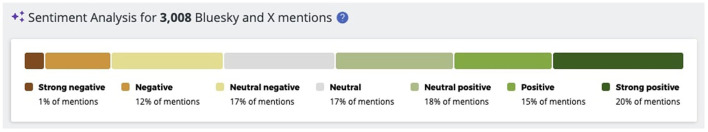
Example of sentiment distribution for a publication inside Altmetric.com.

This study will serve as the foundation for the Beta release of sentiment analysis in Altmetric Explorer, a tool that tracks all research's online attention with insights (https://altmetric.com/). As part of this effort, we plan to analyze sentiment across all X/Twitter and Bluesky posts that mention any research outputs. Figure 4 presents the early prototype of how a visualization analyzing a publication sentiment might look inside Altmetric Explorer. In the initial phase, users will have access to detailed sentiment scores for individual mentions, as well as aggregated results presented with corresponding percentages. This feature will enable users to understand how sentiment is distributed and contributes to an overall assessment of a research output.

Additionally, we aim to provide users with a user-friendly interface that visualizes the collective sentiment of a set of papers based on their search criteria. For instance, if a user searches for all publications from a specific journal over the past year, they will receive an overview indicating whether the overall sentiment is positive (e.g., 55%), which can serve as a valuable tool to understand public and professional perception and gauge performance, among other things. To further enhance usability, we intend to include options for filtering sentiment posts by score. This functionality will allow users to quickly identify and examine the most positive or negative mentions of their research outputs. Along with developing the framework, we will create a suite of evaluation tests based on the results of our study. These tests will be used to systematically evaluate the impact of any modifications to the sentiment analysis model. Through iterative refinement of both the prompt and the model guided by these evaluations, we aim to ensure that changes enhance its overall effectiveness. Future refinements could focus on optimizing precision while preserving the high recall rate, potentially through post-processing methods such as confidence scoring, threshold tuning, or weighted loss functions that balance misclassification costs in a multi-class setting.

## 5 Conclusion

This study demonstrated that LLMs can help move beyond surface-level metrics to understand better how research is framed, used, and discussed in public digital spaces. By focusing on sentiment toward the use of research, rather than just the emotional tone of posts, we developed a bespoke classification system that captures a wider spectrum of engagement, from endorsement and support to critique and skepticism. Social media platforms are not passive channels of dissemination; they are dynamic environments where research is mobilized to persuade, challenge, or signal credibility. Our findings showed that AI can play a critical role in uncovering how research is received and repurposed in these contexts, offering a more nuanced understanding of attention and influence in our society.

## Data Availability

The data analyzed in this study is subject to the following licenses/restrictions: The main dataset used for this analysis is available in Appendix 1 and can be tested in any social media post for sentiment analysis of the use of research outputs. Other requests should be directed to the corresponding author.
